# Lipids in Mitochondrial Macroautophagy: Phase Behavior of Bilayers Containing Cardiolipin and Ceramide

**DOI:** 10.3390/ijms24065080

**Published:** 2023-03-07

**Authors:** Yaiza R. Varela, Emilio J. González-Ramírez, Marina N. Iriondo, Uxue Ballesteros, Asier Etxaniz, Lidia Ruth Montes, Félix M. Goñi, Alicia Alonso

**Affiliations:** Instituto Biofisika (UPV/EHU, CSIC), and Department of Biochemistry and Molecular Biology, University of the Basque Country, E-48940 Leioa, Spain

**Keywords:** ceramide, cardiolipin, nanodomains, autophagy, LC3/GABARAP

## Abstract

Cardiolipin (CL) is a key lipid for damaged mitochondrial recognition by the LC3/GABARAP human autophagy proteins. The role of ceramide (Cer) in this process is unclear, but CL and Cer have been proposed to coexist in mitochondria under certain conditions. Varela et al. showed that in model membranes composed of egg sphingomyelin (eSM), dioleoyl phosphatidylethanolamine (DOPE), and CL, the addition of Cer enhanced the binding of LC3/GABARAP proteins to bilayers. Cer gave rise to lateral phase separation of Cer-rich rigid domains but protein binding took place mainly in the fluid continuous phase. In the present study, a biophysical analysis of bilayers composed of eSM, DOPE, CL, and/or Cer was attempted to understand the relevance of this lipid coexistence. Bilayers were studied by differential scanning calorimetry, confocal fluorescence microscopy, and atomic force microscopy. Upon the addition of CL and Cer, one continuous phase and two segregated ones were formed. In bilayers with egg phosphatidylcholine instead of eSM, in which the binding of LC3/GABARAP proteins hardly increased with Cer in the former study, a single segregated phase was formed. Assuming that phase separation at the nanoscale is ruled by the same principles acting at the micrometer scale, it is proposed that Cer-enriched rigid nanodomains, stabilized by eSM:Cer interactions formed within the DOPE- and CL-enriched fluid phase, result in structural defects at the rigid/fluid nanointerfaces, thus hypothetically facilitatingLC3/GABARAP protein interaction.

## 1. Introduction

Autophagy is an intracellular degradation pathway conserved in all eukaryotes. A frequent form of autophagy called macroautophagy consists of the degradation of damaged whole cell organelles, misfolded cytosolic proteins, or invasive microorganisms. Macroautophagy involves the formation of a double-membrane structure, the phagophore, and its subsequent maturation to form an autophagosome (AP) that fuses with lysosomes where the autophagosomal components are degraded and potentially recycled [1]. Several cargo-specific autophagy processes have been reported, including the specific removal of mitochondria known as mitophagy. Beyond quality control, mitophagy is required for the steady-state turnover of mitochondria, for the adjustment of mitochondrion numbers to changing metabolic requirements, and during specialized developmental stages in mammalian cells [2]. In the context of this work, ‘autophagy’ will be used as a short synonym for macroautophagy.

The involvement of several membranous systems in autophagy explains the important role of several lipids in the process. Significant examples include the important role of phosphatidyl inositol 3-phosphate in autophagosome formation [3] and the obligatory covalent binding of the LC3/GABARAP autophagy protein family to phosphatidyl ethanolamine in the nascent phagophore as a step in autophagosome formation [4,5]. Previous work from this laboratory has pointed out the role of lipid geometry and membrane curvature in autophagosomal expansion mediated by LC3/GABARAP, the human equivalents of the yeast protein Atg8 [6], and the effects of both lipid geometry and lipid charge on the binding of human ATG3 to lipid membranes [7]. In fact, many stages of AP generation can be rationalized in terms of curvature, including both the molecular geometry of lipids (‘intrinsic curvature’) and the overall mesoscopic curvature of the whole membrane, as observed with microscopy techniques (see the review in [8]).

Further studies from our group include the role of cardiolipin (CL)-LC3/GABARAP interactions in mitophagy [9,10] and more recently, the capacity of ceramide (Cer) to enhance the binding of LC3/GABARAP autophagy proteins to CL-containing membranes in mitophagy, the specific degradation of mitochondria via autophagy [11,12]. A substantial part of these investigations has been carried out on model membranes of defined lipid compositions. The importance of the results of model membranes in the understanding of cell membranes cannot be overemphasized.

The present contribution arises from our recent studies described in [11,12]. Those investigations were based on bilayers composed of egg sphingomyelin (eSM), dioleoyl phosphatidyl ethanolamine (DOPE), and CL. Cer was included in the mixture: it was either added in the preparation steps or generated in situ via the enzymatic hydrolysis of SM with a sphingomyelinase [13]. In eukaryotes, CL is almost exclusively located in the mitochondrial inner membrane, while Cer appears mainly in the plasma membrane at the onset of the sphingolipid signaling pathway, leading to apoptosis. Even if the relevance of Cer in the binding of LC3/GABARAP to membranes was suggested [14], CL and Cer are rarely found together in membranes. Indeed, this rarity may perhaps be the reason for the scarcity of physical–chemical studies with these mixtures. However, recent work by Vos et al. [15] has shown that Cer accumulation induces mitophagy and impairs β-oxidation under conditions of PINK1 deficiency, which is related to early-onset Parkinson’s disease. This explains the interest in the CL/Cer system for model membrane autophagy studies.

CL is unique among phospholipids in possessing four fatty acyl chain residues. It also has two net negative charges at neutral pH whereas the most common species in mitochondria (tetralinoleoyl) possesses a negative intrinsic curvature [16,17]. Ceramide in turn is a non-phosphoryl-containing sphingolipid, characterized by an extremely low polarity [18]. The data by Varela et al. [11,12] showed that, when incorporated into a matrix of egg SM and DOPE, CL was essential for LC3/GABARAP interaction with these bilayers and that Cer markedly increased binding. Moreover, giant unilamellar vesicles (GUV) examined under confocal fluorescence microscopy revealed that Cer segregated laterally into very rigid domains, while GABARAP bound only the more fluid regions, suggesting that the enhancing role of ceramide is exerted by the minority of ceramide molecules dispersed in the fluid phase.

The above data and hypotheses highlight the importance of a detailed examination of the phase properties of phospholipid mixtures containing CL and Cer. The present contribution intends to fill this gap, with the combined use of differential scanning calorimetry (DSC), confocal microscopy of fluorescence stained GUV, and atomic force spectroscopy (AFM) of supported lipid bilayers (SLB).

## 2. Results

Ceramide-induced phase segregation in the various membrane model systems was studied using differential scanning calorimetry (DSC), confocal microscopy, and atomic force microscopy (AFM). DSC provided information about the thermodynamic parameters of the different samples. The gel–fluid mid-point transition temperature T_m_ indicates the stability of the gel phase: higher T_m_ values are related to more stable gel phases. The endotherm width-at-half-height T_1/2_ is related to the cooperativity of the thermotropic transition: the lower the T_1/2_ values, the higher the cooperativity. ∆H is related to the heat exchange in the transition and it was calculated from the endotherm area (specifically the integration of C_p_ vs. T) [16]. Confocal microscopy of giant unilamellar vesicles (GUV) was used to confirm the coexistence of different lipid phases in a given sample using Rho-PE, a fluorescence probe that partitions out of liquid-ordered phases and into liquid-disordered phases [19]. The topography of the samples, in the form of supported lipid bilayers (SLB), was analyzed using AFM once the sample was supported on a flat mica surface. Proper lipid mixing in the bilayers could be confirmed by AFM due to the absence of lipid aggregates. Then, nanomechanical properties such as the breakthrough force (F_B_) of the different phases could be obtained using AFM in the force spectroscopy mode.

### 2.1. eSM-Based Samples

**Figure 1:**
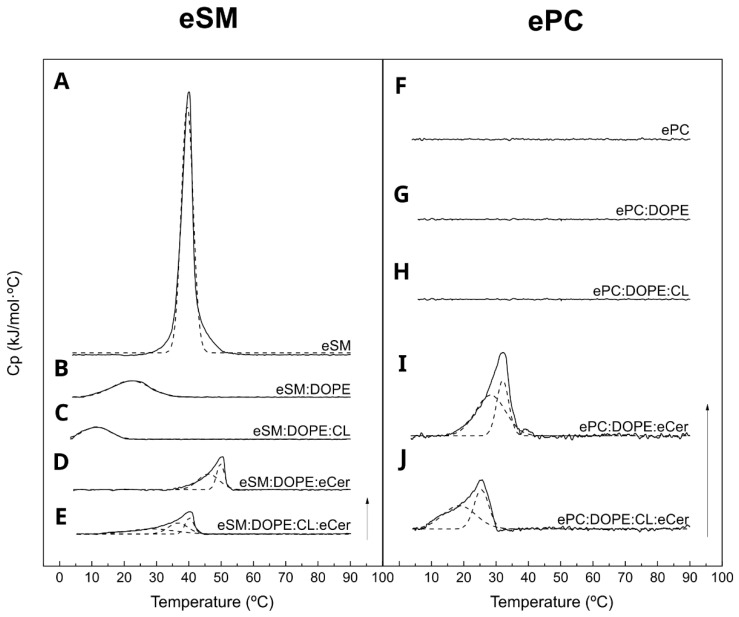
DSC thermograms of lipid mixtures dispersed in excess water. eSM (**A**); eSM:DOPE (50:50) (**B**); eSM:DOPE:CL (33:33:33) (**C**); eSM:DOPE:eCer (40:50:10) (**D**); eSM:DOPE:CL:eCer (23:33:33:10) (**E**); ePC (**F**); ePC:DOPE (50:50) (**G**); ePC:DOPE:CL (33:33:33) (**H**); ePC:DOPE:eCer (40:50:10) (**I**); and ePC:DOPE:CL:eCer (23:33:33:10) (**J**). Representative thermograms of three independent preparations. The dotted lines correspond to Gaussian fittings of the thermograms in solid line. Arrow: 1 kJ/mol °C.

Five eSM-based samples were measured and all showed a gel–liquid endothermic transition when analyzed by DSC. Pure eSM was the simplest sample: it showed a sharp phase transition with a T_m_ around 40 °C and, as expected from a pure lipid, the highest enthalpy value (ΔH = 31.5 kJ/mol·°C), the highest change in transition entropy (ΔS = 100.7 ± 0.8 J/mol·°C), and the lowest T_1/2_ value (4.1 °C) (Figure 1A, Table 1). The binary sample composed of eSM and DOPE at an equimolar ratio and existed in a liquid phase at room temperature. In this case, the presence of DOPE significantly decreased the T_m_ and enthalpy values to 21.8 °C and 5.4 kJ/mol·°C, respectively (Figure 1B, Table 1), in agreement with previous studies [20]. Moreover, T_1/2_ increased, indicating a lower cooperativity in this sample. When the ternary samples were analyzed, two different behaviors were observedwith CL and Cer, respectively. The addition of polyunsaturated lipid CL caused a further decrease in T_m_ and ∆H (Figure 1C), while the addition of the high-melting Cer markedly increased them (Figure 1D). Notably, Cer addition gave rise to higher lipid cooperativity (Figure 1D, Table 1). The sample containing both CL and Cer, eSM:DOPE:CL:eCer (23:33:33:10), showed intermediate values between the two ternary samples previously analyzed (Figure 1E, Table 1). Furthermore, the absence of an endothermic transition around 80–90 °C in these thermograms confirmed the full incorporation of ceramide into the mixtures (Figure 1D,E), with pure Cer giving off endotherm signals in that T range [21].

**Figure 2:**
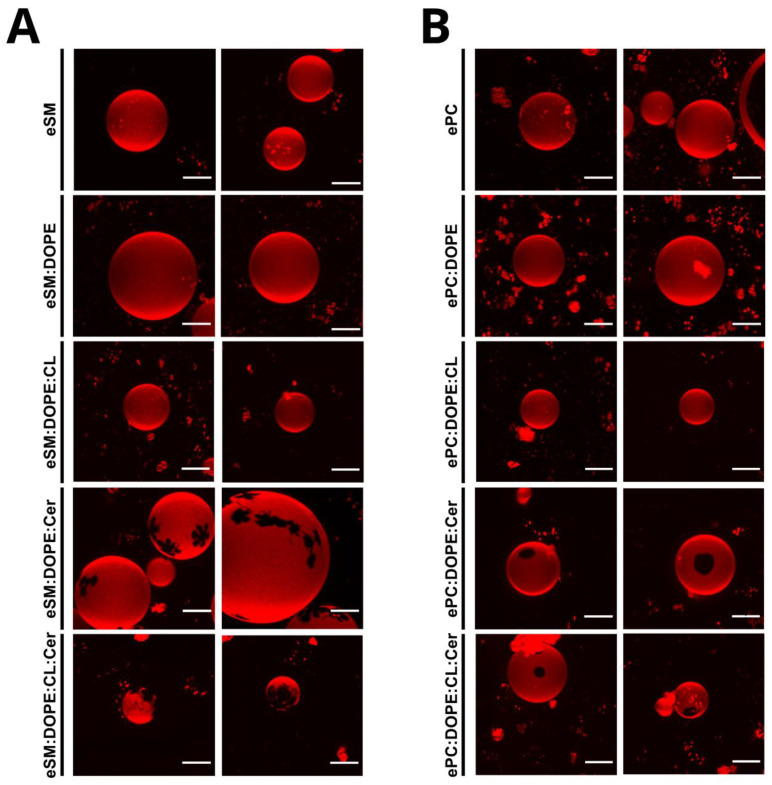
3D-Confocal microscopy of GUV of different lipid mixtures. Rho-PE staining. eSM-based samples (**A**) and ePC-based samples (**B**). Scale bar = 10 µm. Representative fields of 10–30 GUV per preparation.

**Figure 3:**
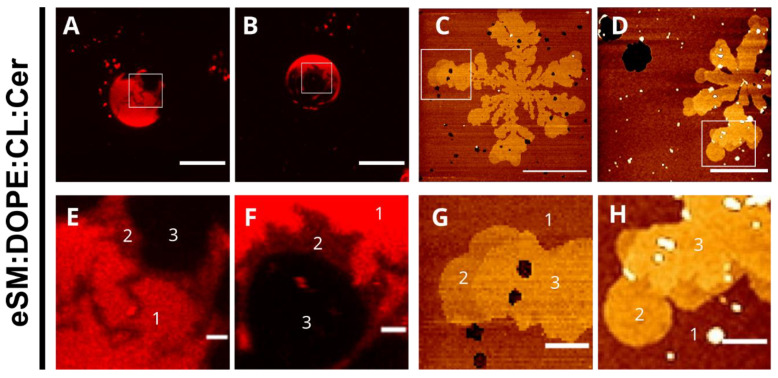
Magnified fields to facilitate the observation of coexisting two rigid phases in eSM:DOPE:CL:eCer (23:33:33:10) mixtures. The bottom images correspond to the white boxes in the top images but observed with a higher magnification. The top images are taken from Figure 2A bottom (**A**,**B**); and not shown (**C,D**). Confocal microscopy (**A**,**B**,**E**,**F**). Bars: A and B, 10 μm, and E and F, 1 μm. AFM (**C**,**D**,**G**,**H**). Bars: C and D, 5 μm, and G and H, 1 μm. Phases in the amplified fields: 1, continuous fluid phase; 2, minor rigid domain; 3, main rigid domain.

Rho-PE is a fluorescent probe that partitions preferentially into less ordered phases. It was used with GUV in order to observe the potential existence of gel phases (Figure 2A). Samples without ceramide, eSM, eSM:DOPE (50:50), and eSM:DOPE:CL (33:33:33), showed fully stained GUV in all analyzed cases, which confirmed the absence of segregated phases. However, the eSM:DOPE:eCer (40:50:10) composition showed two different phases: one fully stained and one totally depleted of Rho-PE, corresponding to a fluid and a gel phase, respectively. Moreover, eSM:DOPE:CL:eCer (23:33:33:10) presented an additional phase, as compared to the previous sample, which was partially stained with the fluorophore and located between the two other phases (Figure 2A, bottom, Figure 3A,B,E,F). Notably, in general the photo selection effect [22,23] led to an apparent less intense Rho-PE staining in the GUV polar regions.

**Figure 4:**
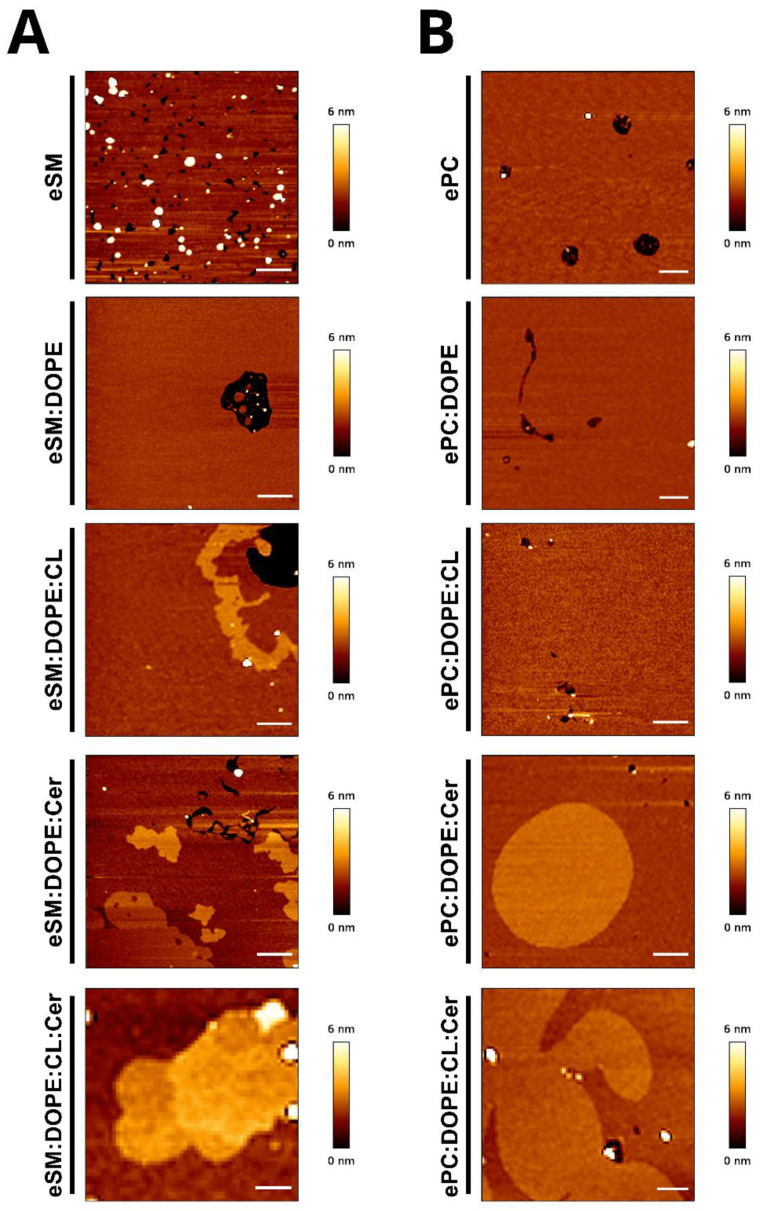
AFM topology of different lipid mixtures. eSM-based samples (**A**) and ePC-based samples (**B**). 3 µm × 3 µm images, scale bar = 500 nm. Representative images of three independent preparations.

In order to characterize the different phases present in the sample, SLBs were constructed and examined by AFM (Figure 4). eSM, eSM:DOPE (50:50), and eSM:DOPE:CL (33:33:33) showed homogenous topologies. The ternary sample with ceramide, eSM:DOPE:eCer (40:50:10), showed a continuous phase together with segregated domains. The latter had a higher thickness than the continuous phase, as shown in Table 2. The most complex sample, eSM:DOPE:CL:eCer (23:33:33:10), confirmed the presence of three different phases, as observed with GUV. Two of those appear as flower-like segregated phases with a higher thickness than the continuous phase. The third was apparent mainly in the distal parts of the flower-like domains (domain within a domain), and its thickness was intermediate between the previous two (Figure 3C,D,G,H). Force spectroscopy data (Table 3) showed that, as expected, segregated phases were stiffer than the continuous phase. Furthermore, the thickest of the ceramide-enriched domains in eSM:DOPE:CL:eCer (23:33:33:10) were stiffer than the domains in eSM:DOPE:eCer (40:50:10). Overall, our results show a clear effect of eCer on the nanomechanical properties of the lipid bilayers containing CL.

### 2.2. ePC-Based Samples

In order to assess the relevance of the sphingosine moiety of eSM in the above results, parallel measurements were performed in which ePC was used instead of eSM. Samples without ceramides, ePC, ePC:DOPE (50:50), or ePC:DOPE:CL (33:33:33) did not show a transition temperature given that all these lipids were in a fluid phase above 0 °C (Figure 1F–H). However, samples with ceramides, ePC:DOPE:eCer (40:50:10), and ePC:DOPE:CL:eCer (23:33:33:10) exhibited a transition temperature with T_m_ around 32 °C and 26 °C, respectively (Figure 1I,J, Table 1). Both sample types present a lower enthalpy value and a higher T_1/2_ than their eSM-based counterparts. This was expected due to the substitution of a lipid in the gel phase at room temperature (eSM) for one that exists in a fluid phase under those conditions (ePC).

When Rho-PE was used to identify the presence of ceramide-enriched areas in GUV, the observed behavior was compatible with the calorimetric measurements (Figure 2B). Samples without ceramide were uniformly stained with Rho-PE due to the fluid state of the lipid mixture at room temperature. Both ceramide-containing samples showed domains devoid of fluorescence. The contours of these domains were smoother than in the case of the eSM-based samples. The four-component ePC-based sample showed just one kind of segregated phase (Figure 2B), in contrast with the equivalent preparation based on eSM.

AFM analysis of non-ceramide-containing samples revealed a homogenous phase in all the experiments performed. Samples with ceramide showed ceramide-enriched segregated domains which were thicker than the continuous phase (Figure 4B, Table 2). Force spectroscopy data indicated that the segregated phases were stiffer than the continuous phase, as expected (Table 3). As was the case for the eSM-based samples, the segregated domains in the four-component ePC-based mixture were more rigid than those in the Cer-containing ternary sample, even when the T_m_ of the whole mixture was lower (Table 1). It is additionially noteworthy that observation of the quaternary sample could be perturbed by the fact that the temperature of analysis (23 °C) fell within the transition temperature range of the sample (Figure 1J). This gave rise to segregated phases being laterally displaced in the bilayer plane during the analysis (not shown).

## 3. Discussion

In previous work [11,12], two different mechanisms by which ceramide increases LC3/GABARAP binding to CL-containing bilayers were proposed: either Cer present in the liquid phase was mainly responsible or the presence of ceramide in the membrane produced a lipid restructuring, which favors the protein binding. In order to elucidate this mechanism, differential scanning calorimetry (DSC), confocal microscopy, and atomic force microscopy (AFM) were performed.

### 3.1. Formation of CL-Containing Bilayers

Among the lipids used in this work, both SM and PC give rise to lamellar structures in excess water. However, the presence of DOPE, CL, and Cer in the membrane can generate a highly negative curvature and favor the formation of inverted hexagonal (H_II_) phases [8,24,25]. This happens because the latter three lipids have a conical shape in the Israelachvili nomenclature [26], meaning that the cross-sectional area of their headgroups is smaller than that of their acyl chains. Moreover, ceramide is a highly hydrophobic molecule that tends to occupy the space at the level of the acyl chains, largely avoiding its interaction with the polar solvent (see structural details in [18,27]). Moreover, ceramide is a highly hydrophobic molecule which tends to occupy the space in the boundary between the acyl chains and the polar headgroup, which is large enough to avoid its interaction with the polar solvent, giving rise to ceramide-enriched areas [27]. The comparative use of three very different techniques provides reliable information with small discrepancies, allowing a better understanding of the interaction between these lipids.

To the authors’ knowledge, there is little information on SLB with high (>20 mol%) concentrations of cardiolipin. Unsay et al. [28] studied the formation and dynamics of CL-containing bilayers with the highest concentration of CL in the sample being 20 mol%. In SLB preparation, the presence of divalent ions such as Mg^2+^ or Ca^2+^ favors the formation of properly extended SLB [29]. However, the presence of the two negative charges of cardiolipin and divalent cations in the buffer gives rise to non-lamellar structures [30]. To avoid the formation of undesired structures, a rinsing process was added to the SLB preparation protocol to eliminate the free divalent cations in the solution. In this way, the formation of bilayers that did not cover the entire substrate surface was made possible. These lipid-depleted areas allowed for measuring bilayer thicknesses and performing proper controls for force-spectroscopy measurements [31].

Regarding the bilayer thickness, Unsay et al. [28] provided several hypotheses as to how the presence of CL could affect the lipid bilayer structure. CL insertion into a PE-based bilayer [POPE:CL (80:20)] caused variations in thickness and hence several segregated phases were observed depending on the temperature [32]. In our experiments, no differences in height were detected upon the addition of CL; bilayer thickness was around 5 nm for samples with and without CL (Table 2). Recent experiments based on simulations concluded that CL was able to induce changes in the lipid packing of the samples, but not in the bilayer thickness [23]. Assuming good lipid mixing in both cases, the different results between Domenech’s [32] and ours may be attributed, to the different fluidity of the POPE and DOPE bilayers, with the more unsaturated DOPE also being more miscible with CL.

AFM in the force spectroscopy mode is a useful tool to study lipid packing through measurements of the breakthrough force (F_B_). This is the force needed to pierce the bilayer and can be related to the nanomechanical stability of the membrane. A known force is applied to a specific area of the sample, and when the tip crosses the bilayer a jump in the force curve appears. When DOPE and CL are added to eSM, these lipids tend to destabilize the gel phase and a notable decrease in the breakthrough force value from 20 nN for pure eSM to 3 nN for eSM:DOPE:CL (33:33:33) is observed. This effect is not as pronounced in the case of ePC-based samples since ePC is in a liquid phase at room temperature. Unsay et al. [28] also used the F_B_ value in their studies. They concluded that CL addition generated a “double jump” in the curve due to the instability produced by this phospholipid, and analyzed how different CL concentrations affected the nanomechanical resistance of lipid bilayers. However, the meaning of a double jump has been widely debated without reaching a unanimous conclusion. For example, Jimenez-Rojo et al. [21] reported on a double jump event in the force curves of pure pSM. Alessandrini and Facci [33] explained that this phenomenon could be related to interleaflet coupling which is affected by the support due to the generation of vertical asymmetries, while Seeger et al. [34] pointed to the different preparation procedures and the ionic strength of the solution as possible reasons for this phenomenon.

### 3.2. Lipid Redistribution Caused by Ceramide in Cardiolipin-Containing Vesicles

With the aim of studying the effect of ceramide in these membrane models systems, ternary samples were prepared. Ceramide is known to segregate laterally into highly ordered gel-like domains [35,36]. eSM:DOPE:Cer (33:33:33) shows an increase in T_m_ as compared with pure eSM and eSM:DOPE samples. The increase can be related to the higher stability of the Cer-rich gel phase. ePC:DOPE:Cer (33:33:33) shows a gel to liquid phase transition about 20 °C below that of eSM:DOPE:Cer due to the low (sub 0 °C) T_m_ of ePC (Figure 1). The relative cooperativities suggested by T_1/2_ (Table 1) confirm the stronger interaction of ceramide with eSM than with ePC. As previously described, GUV and AFM topography images provide consistent results. Ceramide segregated areas in eSM-based samples show flower-shaped domains because of dipole–dipole repulsion is the predominant force, while in ePC-based samples, as the domain line tension is the strongest force, non-branched domains are predominant [37]. Furthermore, ceramide-enriched areas show a higher stiffness in eSM-based samples than in ePC-based samples, as expected.

The most complex samples, in which both Cer and CL are present, contain ceramide-enriched areas even when the Cer molar concentration decreases from 33 to 10 mol%. As mentioned above (Section 2.2), ceramide-rich domains in ePC-based bilayers could diffuse laterally during the study because the temperature of analysis (23 °C) was close to the transition temperature of the mixture (~26 °C) (Figure 1J). eSM:DOPE:CL:Cer (23:33:33:10) shows two different segregated phases, observed with both confocal microscopy and AFM (Figure 2, Figure 3 and Figure 4). These segregated phases present a different degree of Rho-PE staining in GUV. Both appear as “domains within domains” when they are analyzed using atomic force microscopy, a phenomenon that has been previously observed in samples involving SM:Cer interactions [38]. Such differentiated segregated phases had escaped our attention when preparing our previous contribution [11]. The possibility that some of them correspond to out-of-register domains consisting of two leaflets of different lipid composition deserves some further study.

In relation to the lipid packing in the quaternary-sample segregated domains, they exhibit the highest breakthrough force values obtained in our experiments, both in eSM- and ePC-based bilayers (Table 3). However, the overall stability of these gel phases, as indicated by the T_m_ values, is lower than that of the ternary samples (Figure 1). This discrepancy can be explained based on the presence of CL: studies based on simulations have shown how the addition of CL increases the area per lipid in bilayers composed of PC and PE [39,40]. CL is composed of two polyunsaturated phosphatidylglycerol moieties connected by a single bridging glycerol group and it is easier for it to settle into the liquid phase rather than in the gel phase. The presence of CL in the liquid phase can produce the displacement of the other lipids present in the sample to the most rigid phase. It should be noted in this respect that when a phase is enriched in a specific lipid, this does not exclude the likelihood that all lipids are present in different amounts in every phase.

The existence of two segregated phases in eSM:DOPE:CL:Cer (23:33:33:10) could be justified by the presence of high concentrations of ceramide in both phases. However, force spectroscopy data point to another option. The breakthrough force obtained for the thinner segregated phase is quite similar to that obtained for eSM:DOPE (50:50) (Table 3), thus pointing to a similar lipid composition, while the second segregated phase would be a ceramide-enriched phase.

The above data were obtained in model membranes whose composition differs from the average mitochondrial membrane composition. Indeed, Cer and SM are minority lipids in mitochondria. However, it is possible that micro/nanodomains are enriched in those lipids and that the Cer concentration may be increased under PINK1 deficiency [15]. Our results show that such small domains can indeed exist under certain conditions. The affinity of Cer for SM is such that, even at small concentrations, these lipids are expected to interact and segregate laterally [36]. Our model membranes are not intended to represent the whole mitochondrial membrane, but rather those micro/nanodomains.

### 3.3. The Relevance of Ceramide Miscibility for LC3/GABARAP Binding to Membranes

In our recent article [11,12], how the binding of LC3/GABARAP autophagy proteins to eSM:DOPE bilayers containing CL was enhanced by the presence of Cer was shown. Fluorescence microscopy of GUV revealed that in eSM:DOPE:CL:Cer mixtures, Cer gave rise to laterally segregated and highly rigid domains, in agreement with previous observations [18,35,36,37,38]. The results in this paper (Figure 2, Figure 3 and Figure 4) show the same Cer-rich rigid domains both in GUV and in supported lipid bilayers. Moreover, Varela et al. [11,12] found that, when one of the LC3/GABARAP proteins was labeled with the fluorescent probe Alexa 633, confocal fluorescence revealed that the protein bound the fluid ceramide-poor regions in the GUV preferentially. In summary, protein binding to CL-rich domains was facilitated by two independent factors, namely fluidity (provided by CL) and the presence of Cer. Moreover, LC3/GABARAP proteins have a specific affinity for CL. The minority fraction of Cer in the fluid domains appeared to be responsible for the enhanced binding. This Cer fraction had been estimated at 2–5 mol% of the total lipid in those domains [35,41]. Thus, Cer helps in protein binding to the bilayers but, in the rigid Cer-rich domains, lipid density is too high to allow protein docking. Consequently, Cer enhances binding only when it is dispersed (in a small amount) in the fluid phase. In our previous paper [11,12], it was suggested that the diluted Cer in the fluid phase could contribute to protein binding in at least two ways: (i) a Cer-induced local perturbation of nanometric dimensions and of physical properties in CL-rich domains (see [42,43] for a discussion on nanodomains) and (ii) given that both CL and Cer are ‘negatively curved lipids’ [8] the putative formation of a transient non-lamellar intermediate that would facilitate protein insertion [44] could not be excluded.

The results in the present paper describe the behavior of bilayers containing CL and Cer, thus confirming and extending the previous data [11,12]. The most relevant novel observations are the presence of three segregated phases in the samples containing eSM:DOPE:CL:Cer which are detected by DSC (Figure 1A, Table 1), confocal microscopy (Figure 2 and Figure 3), and AFM (Figure 3 and Figure 4, Table 2 and Table 3). The fact that the three phases are distinguished using three fundamentally different techniques adds credibility to these results. The very wide transition in Figure 1J means that both the experimental T for GUV and AFM (23 °C) and the human physiological T (37 °C) fall within the transition, i.e., the domain coexistence is expected to occur in all cases. The observation of phase segregation at the micrometer scale, as shown above, does not immediately explain the origin of LC3/GABARAP binding to the fluid domains, nor does it guarantee the formation of equivalent phase separation at the nanometer scale. However, recent lines of evidence suggest that phase separation at the micro-scale should be a good indication for an equivalent nano-scale segregation. In particular, on the basis of experimental data arising from multiple four-component phase diagrams, Feigenson [45] concluded that bilayer physical and chemical properties measured from macroscopic domains of coexisting liquid-disordered and liquid-ordered phases should be good approximations for the properties of coexisting nanoscopic domains. We may thus propose that the data in this paper are compatible with the existence of Cer-enriched nanodomains dispersed in an overall fluid bilayer. Cer nanodomains would facilitate protein binding because proteins often show a preference for the structural defects appearing in the bilayer at the interfaces between ordered and disordered domains [46,47].

The observation (Figure 1, Figure 2 and Figure 4) that only two phases are seen in the ePC-based samples is noteworthy. This is probably related to the fact that SM and Cer, but not PC or Cer, exhibit a strong interaction and give rise to very rigid, detergent-resistant, and segregated domains [36,48]. The AFM images in Figure 4 show fractal or irregular contours for Cer-containing, eSM-based samples, suggestive of a rigid–fluid domain interface. Figure 3 also reveals at least two rigid phases for the quaternary mixture. In contrast, the domains in ePC-based bilayers are smooth. This is probably an indication that the line tension of the micron-sized ePC-based domains is high enough to prevent or decrease nanodomain formation, while the strong interaction of SM:Cer complexes would overcome the line tension limitation in micron-sized domains and allow for the generation of nanodomains within a continuous fluid phase.

It can be concluded that the membrane model systems included in this work may explain the mechanism by which ceramide increases LC3/GABARAP binding to CL-containing bilayers. Cer appears to cause a reorganization of the membrane lipids. The displacement of lipids such as SM to rigid domains together with Cer would generate nanodomains dispersed within CL-enriched fluid areas, and the rigid-fluid interfaces would be preferential areas for LC3/GABARAP protein binding. This situation might have a parallel in the outer mitochondrial membrane, in which CL externalization acts as an autophagic stimulus to promote damaged mitochondria recognition by LC3/GABARAP, thus reinforcing the potential role of ceramide in mitochondrial autophagy (mitophagy).

## 4. Materials and Methods

### 4.1. Chemicals

Sphingomyelin (egg, cicken) (eSM, 860061); L-α-phosphatidylcholine from hen egg yolk (ePC, 840051); 1,2-dioleoyl-sn-glycero-3-phosphatidylethanolamine (DOPE, 850725); heart bovine cardiolipin; ≈90% tetralinoleoyl (CL, 840012); ceramide (egg, chicken) (eCer, 860051); and 1,2-dioleoyl-sn-glycero-3-phosphatidylethanolamine-N-(lissamine rhodamine B sulfonyl) (Rho-PE, 810150) were purchased from Avanti Polar Lipids, Inc. (Alabaster, AL, USA). Methanol and chloroform were obtained from Fisher (Suwanee, GA,USA). The buffer solution, unless otherwise stated, was 20 mM piperazine-N,N’-bis(2-ethanesulfonic acid) (PIPES), 1 mM ethylene diamino tetraacetate (EDTA), 150 mM NaCl, pH 7.4. All salts and organic solvents were of analytical grade, supplied by Sigma (St. Louis, MO, USA).

### 4.2. Multilamellar Vesicle (MLV) Preparation

All lipid mixtures are given as mole ratios. Lipid vesicle preparation consists of mixing the desired lipids dissolved in chloroform/methanol (2:1, *v/v*) and drying the solvent under a stream of nitrogen. The removal of undesired organic solvent is ensured by keeping the lipid film under a high vacuum for 60 min. Multilamellar vesicles (MLV) were formed by hydrating the lipid film with the buffer solution at 50 °C.

### 4.3. Differential Scanning Calorimetry (DSC)

The measurements were performed in a nano-DSC (TA Instruments, New Castle, DE, USA). MLV to a final concentration of 1 mM was prepared as described above with a slightly different hydration step: instead of adding the buffer solution in a single step, increasing amounts of the solution were added, helping the dispersion by stirring with a glass rod. Then, the vesicles were homogenized by forcing the sample 50–100 times between two syringes through a narrow tube (0.5 mm internal diameter, 10 cm long) at a temperature above the transition temperature of the lipid mixture. Before loading the MLV sample into the appropriate calorimeter cell, both the lipid and buffer solutions were degassed. In total, 0.5 mL of suspension containing 1 mM total lipid concentration was loaded into the calorimeter, performing 6 heating scans at a 45 °C/h rate, between 0 and 100 °C for all the samples. After measurements were taken, the software provided with the nano-DSC was used to subtract the baseline from the sample scan. Moreover, a sigmoid adjustment was performed to obtain a flat line before and after the transition temperature. Finally, the phospholipid concentration was determined using a phosphorus assay and the thermogram obtained was normalized with the concentration obtained. The different thermodynamic parameters were obtained from the thermograms using the software Origin 7.0 (MicroCal, Malvern, UK). Gaussian fittings of the thermograms were performed to visualize the different components in each endotherm. Further information on calorimetric studies of ceramide-containing samples can be retrieved elsewhere [49].

### 4.4. Confocal Microscopy of Giant Unilamellar Vesicles (GUV)

A lipid stock of the desired GUV composition was prepared at 1 mM in chloroform:methanol (2:1, *v/v*). Labeling was carried out by pre-mixing the desired fluorescent probe (Rho-PE) with the lipids in organic solvent. The fluorescent probe concentration was 0.5 mol% Rho-PE. In total, 20 μL of the stock was placed on indium tin oxide (ITO) coated glass electrodes (10 μL on each conducting surface) and kept under vacuum for 1 h to remove solvent traces [22]. Then, 300 mM sucrose was added to the lipid mixture. The electroformation protocol consisted of 10 Hz, 1 Vrms during 90 min, and ≈10 °C above the transition temperature of the lipid mixture. Finally, GUV were transferred to the visualizing chamber, where 50 mM Tris-HCl and150 mM NaCl pH 7.5 buffer were added. Images were acquired on a Leica SP5 confocal microscope with a 63x Water Planar Apochromat 1.2 NA objective at 23 °C. The excitation and emission wavelengths used for Rho-PE were 514 nm and 580–600 nm, respectively. A slow cooling, performed in the dark, from the formation temperature to room temperature, was needed to observe large enough domains by fluorescence microscopy [23]. No difference in domain size, formation, or distribution was detected in the GUV during the observation period or after laser exposure.

### 4.5. Supported Lipid Bilayer (SLB) Formation

MLV are prepared as described above, introduced in a FB-15049 (Fisher Scientific Inc., Waltham, MA, USA) bath sonicator, and kept at 80 °C for 1 h. In this way, a proportion of small unilamellar vesicles (SUV) were generated. SLB were prepared on high V-2 quality scratch-free mica substrates (Asheville-Schoonmaker Mica Co., Newport News, VA, USA) previously attached to round 24 mm glass coverslips by the use of a two-component optical epoxy resin (EPO-TEK 301-2FL, Epoxy Technology Inc., Billerica, MA, USA). SLB were obtained following the vesicle adsorption method [50,51]. Thereafter, 80 μL sonicated vesicles and 120 μL assay buffer containing 3 mM CaCl_2_ were added onto previously prepared 1.2 cm^2^ freshly cleaved mica substrate mounted onto a BioCell coverslip-based liquid cell for atomic force microscopy (AFM) measurements (JPK Instruments, Berlin, Germany). It has been described that the addition of divalent cations such as Ca^2+^ or Mg^2+^ enhances the vesicle adsorption process onto mica substrates [29]. The final lipid concentration was 150 μM. Vesicles were left to adsorb and extend for 30 min while the sample temperature was kept at 60 °C. In order to avoid sample evaporation and ion concentration, the buffer was constantly exchanged with assay buffer without CaCl_2_ at 60 °C for the remaining time. An additional 30 min were assigned for the samples to equilibrate at room temperature. The non-adsorbed vesicles were discarded by washing the samples 10 times with assay buffer without CaCl_2_ in order to remove remaining Ca^2+^ cations which drastically affect the breakthrough force (F_B_) results of lipid bilayer nanoindentation processes [52]. The efficiency of repeated rinsing to obtain proper and clean supported lipid bilayers has been reported [53]. This extension and cleaning procedure allowed the formation of bilayers that did not cover the entire substrate surface. The presence of lipid-depleted areas helped with the quantification of bilayer thicknesses and the performance of proper controls for force-spectroscopy measurements. Finally, the BioCell was set to 23 °C to start the AFM measurements.

### 4.6. Atomic Force Microscopy (AFM) Imaging

The topography of the supported lipid bilayer was studied in an UltraSpeed AFM (JPK Instruments, Berlin, Germany) using the ‘QI Mode’, an imaging mode that performs force curves simultaneously at a low force. This ensures a lower tip-sample interaction than other methods available which preserves the tip and the sample from damage. For proper measurements, the AFM was coupled to a Leica microscope and mounted onto an anti-vibration table and inside an acoustic enclosure (JPK Instruments). The BioCell liquid sample holder (JPK Instruments) was used in order to control the assay temperature at 23 °C. V-shaped MLCT Si_3_N_4_ cantilevers (Bruker, Billerica, MA, USA) with nominal spring constants of 0.1 or 0.5 N/m were used for bilayer imaging, obtaining 256 × 256 pixel images through ‘QI Mode’. Images were line-fitted using the JPK Data Processing software.

### 4.7. Force Spectroscopy

The above-described V-shaped MLCT Si_3_N_4_ cantilevers were individually calibrated in a lipid-free mica substrate in assay buffer using the thermal noise method. After proper bilayer area localization by AFM topography, force spectroscopy was performed at a speed of 1 µm/sec in no less than 500 × 500 nm bilayer areas in the form of 10 × 10 or 12 × 12 grids. Force steps were determined for each of the indentation curves as reproducible jumps within the extended traces. The resulting histograms were generated from at least 3 independent sample preparations with at least 3 independently calibrated cantilevers (n = 150–800). Control indentations were always performed in lipid-free areas before and after bilayer indentations to ascertain the formation of a single bilayer.

## Figures and Tables

**Table 1:** Thermodynamic parameters of the gel–fluid transitions, as obtained from DSC experiments. Average values ± SD (n = 3). *a, b*, and *c* refer to the various components of the *total* endotherm when it was decomposed for its proper analysis.

Sample		T_m_ (°C)	T_1/2_ (°C)	∆H (kJ/mol·°C)	∆S (J/(mol·°C)
eSM		39.9 ± 0.1	4.1 ± 0.4	31.5 ± 0.3	100.7 ± 0.8
eSM:DOPE (50:50)		21.8 ± 0.7	14.5 ± 0.8	5.4 ± 0.3	18.8 ± 0.1
eSM:DOPE:CL (33:33:33)		11.6 ± 0.6	10.2 ± 0.7	2.9 ± 0.6	10.1 ± 2.3
eSM:DOPE:eCer (40:50:10)	*total*	50.2 ± 0.6	4.9 ± 0.4	4.8 ± 0.5	14.9 ± 1.4
	*a*	45.9 ± 0.7	9.2 ± 1.8	2.9 ± 0.2	
	*b*	50.0 ± 0.2	4.0 ± 1.7	2.1 ± 0.2	
eSM:DOPE:CL:eCer (23:33:33:10)	*total*	41.2 ± 1.1	7.9 ± 0.6	5.1 ± 0.9	16.1 ± 2.7
	*a*	28.9 ± 0.5	19.5 ± 4.2	2.2 ± 0.1	
	*b*	37.5 ± 0.9	8.5 ± 1.2	2.2 ± 0.2	
	*c*	41.3 ± 1.3	4.3 ± 1.0	1.5 ± 0.3	
ePC		-	-	-	-
ePC:DOPE (50:50)		-	-	-	-
ePC:DOPE:CL (33:33:33)		-	-	-	-
ePC:DOPE:eCer (40:50:10)	*total*	32.2 ± 0.3	12.8 ± 0.1	2.0 ± 0.1	12.2 ± 3.5
	*a*	24.6 ± 3.3	11.8 ± 4.5	2.1 ± 1.0	
	*b*	31.8 ± 0.5	7.3 ± 3.3	2.1 ± 1.4	
ePC:DOPE:CL:eCer (23:33:33:10)	*total*	26.2 ± 0.5	9.2 ± 0.2	3.7 ± 1.1	6.8 ± 0.4
	*a*	19.9 ± 1.3	11.6 ± 1.7	1.3 ± 0.2	
	*b*	25.8 ± 0.5	5.3 ± 1.3	0.8 ± 0.0	

**Table 2:** Bilayer thickness of the different SLB under study. Data expressed as mean ± SD, n = 50. Significance, according to Student’s *t*-test: * *p* < 0.05; ** *p* < 0.01.

Sample	Continuous Phase (nm)	Segregated Phase (nm)	Student’s *t*-test
eSM	4.3 ± 0.3	-	
eSM:DOPE (50:50)	5.0 ± 0.2	-	
eSM:DOPE:CL (33:33:33)	5.0 ± 0.4	-	
eSM:DOPE:eCer (33:33:33)	4.5 ± 0.4	5.6 ± 0.2	*
eSM:DOPE:CL:eCer (23:33:33:10)	5.4 ± 0.4	5.9 ± 0.1 6.2 ± 0.2	- *
ePC	4.9 ± 0.4	-	
ePC:DOPE (50:50)	5.0 ± 0.2	-	
ePC:DOPE:CL (33:33:33)	5.1 ± 0.2	-	
ePC:DOPE:eCer (33:33:33)	4.8 ± 0.2	5.8 ± 0.2	**
ePC:DOPE:CL:eCer (23:33:33:10)	4.9 ± 0.2	5.9 ± 0.2	**

**Table 3:** Bilayer nanomechanical resistance of the different SLB under study. Data expressed as mean ± SD, n = 150–800. Significance, according to Student’s *t*-test: * *p* < 0.05; ** *p* < 0.01; *** *p* < 0.001.

Sample	Continuous Phase (nN)	Segregated Phase (nN)	Student’s *t*-test
eSM	11.1 ± 3.0 20.2 ± 3.2	-	-
eSM:DOPE (50:50)	5.5 ± 1.4	-	-
eSM:DOPE:CL (33:33:33)	3.1 ± 0.4	-	-
eSM:DOPE:eCer (33:33:33)	2.9 ± 1.0	11.3 ± 2.6	**
eSM:DOPE:CL:eCer (23:33:33:10)	2.7 ± 0.6	5.8 ± 1.5 21.6 ± 1.3	* ***
ePC	2.7 ± 0.4	-	-
ePC:DOPE (50:50)	2.6 ± 0.7	-	-
ePC:DOPE:CL (33:33:33)	2.7 ± 0.6	-	-
ePC:DOPE:eCer (33:33:33)	2.0 ± 0.4	5.1 ± 0.7	**
ePC:DOPE:CL:eCer (23:33:33:10)	2.4 ± 1.0	14.1 ± 0.9	***

## Data Availability

Data will be made available on request.
